# Identification of a Metastasis-Related Protein IFI16 in Esophageal Cancer using a Proteomic Approach

**DOI:** 10.7150/jca.59286

**Published:** 2022-03-06

**Authors:** Zhiyu Wang, Bing Sheng, Zichao Wei, Yi Li, Zhihua Liu

**Affiliations:** 1 Guizhou University School of Medicine, Guizhou, 550025, China.; 2 State Key Lab of Molecular Oncology, National Cancer Center/Cancer Hospital, Chinese Academy of Medical Sciences and Peking Union Medical College, Beijing, 100021, China.

**Keywords:** esophageal squamous cell carcinoma (ESCC), metastasis, proteomics, IFI16, FGF1, FGF2

## Abstract

**Background**: Metastasis is the leading cause of the high morality of esophageal squamous cell carcinoma (ESCC), so early monitoring metastasis of esophageal cancer is the key to improve the survival rate of ESCC patients. However, there have not been effective biomarkers for predicting metastasis of ESCC patients,it is an urgent need to identify ESCC metastasis-related proteins.

**Methods**: iTRAQ-based proteomic method was performed in highly metastatic 30M cell established in our previous study and the corresponding parental cells KYSE30.The expression of IFI16 was verified using western blotting and immunohistochemistry (IHC). Then, cck8, transwell assay,mouse metastasis experiments were performed to determine the functional role of IFI16 in esophageal cancer. Finally, RAN-Seq, qpcr, transwell assay were used to investigate the underlying mechanism of IFI16 in esophageal cancer metastasis.

**Results**: The data showed that IFI16 was upregulated in 30M cell compared with KYSE30 cell. IFI16 also increased in ESCC tumor compared with non-tumor tissue. Kaplan-Meier survival curve analysis showed that the relapse-free survival (RFS) of patients with high IFI16 level was worse than that of patients with low IFI16 level (P=0.0449). In addition, IFI16 knockdown did not affect the cell growth, but inhibited ESCC cell migration and invasion in ESCC cells. Moreover, IFI16 knockdown suppressed the lung metastasis of 30M cells in mouse models. Finally, we performed an RNA-Seq assay in IFI16-knocking down 30M cells and identified that knocking down IFI16 downregulated the expression of fibroblast growth factor proteins (FGF1, FGF2 etc.). Furthermore, overexpressing FGF1 and FGF2 rescued the lost of migration and invasion ability of 30M mediated by IFI16 knockdown.

**Conclusion**: Our results demonstrated that IFI16 was a key ESCC metastasis-related protein and played a role in ESCC metastasis through promoting the FGF proteins expression.

## Introduction

Esophageal squamous cell carcinoma (ESCC) is one of the common types of the gastrointestinal tumors with high mortality rate in China 1. The prognosis of patients with esophageal cancer is very poor, and their 5-year survival rate is less than 20% 2, one important reason is the high invasion and metastasis characteristics of esophageal cancer. However, the biological mechanism of esophageal cancer metastasis is still unclear. To develop better diagnosis and treatment approaches, it is important to identify proteins related to ESCC metastasis and understand the molecular basis of ESCC metastasis.

With the exploration of tumor invasion and metastasis, it is found that tumor invasion and metastasis is a complex biological process 3, which can be divided into: reduced adhesion between tumor cells, degradation of extracellular matrix 4, escape of tumor cells from basement membrane, migration into lymphatic vessels or microvessels, immune escape of tumor cells 5, formation of micrometastases and metastatic microenvironment 6. Studies have shown that this complex biological behavior in cancer is an abnormal process regulated by multiple molecules 3, 7. Therefore, finding key molecules for invasion and metastasis is a great significance for further finding of the targets of invasion and metastasis.

The current research on the invasion and metastasis of esophageal cancer mainly focuses on esophageal cancer cell lines with different metastatic capabilities, especially cell lines with the same genetic background but different metastatic capabilities, it's a ideal cell model for studying esophageal cancer metastasis and performing genes differential analysis 8. Here, we directly used the esophageal cancer cell line 30-D (hereinafter referred to as 30M) with high metastasis ability obtained from the KYSE30 cell sieve in our laboratory through the screening model as the research object 9.

The proteome is the functional translation of the genome and the ultimate embodiment of life activities 10. Therefore, quantitative proteomics is one of the hot research fields in the post-genomic era, and it has been widely used in oncology to identify biomarkers with diagnostic and therapeutic potential. In this project, we used iTRAQ (isobaric tags for relative and absolute quantification) combined with liquid chromatography and tandem mass spectrometry to analyze the protein expression of cell 30M with high metastasis capabilities and its parent cell KYSE30 and identify IFI16 as a key molecule involved in cancer metastasis. Our *in vivo* and *in vitro* experiments both demonstrated that IFI16 knockdown inhibited ESCC cell migration and invasion. In addition, ESCC patients with high IFI16 had shorter relapse-free survival (RFS) compared with low IFI16 patients. Finally, we revealed that IFI16 promoted metastasis mainly through increasing FGF proteins expression. Collectively, our results indicated that IFI16 is a key ESCC metastasis-related protein and provided us a possible opportunity for the therapy against ESCC metastasis.

## Materials and Methods

### Cell lines and cell culture

The human ESCC cell lines KYSE30, KYSE180, KYSE450 and other KYSE series were generously provided by Dr. Shimada Y. (Kyoto University, Kyoto, Japan) and cultured in RPMI-1640 medium (Gibco, Carlsbad, CA, USA) supplemented with 10% fetal bovine serum (FBS).The esophageal cancer high metastasis model 30M cells was derived from KYSE30 parental cells in our lab before. The HEK293T cell line was purchased from the American Type Culture Collection (ATCC), preserved in our laboratory, and cultured at 37 °C in 5% CO_2_ in high-glucose Dulbecco's modified Eagle's medium supplemented with 10% FBS (HyClone, South Logan, UT, USA).

### Protein extraction, digestion and labeling with iTRAQ reagents

Cells were sonicated three times on ice using a high intensity ultrasonic processor (Scientz) in lysis buffer (8 M urea, 1% Protease Inhibitor Cocktail), and then centrifuged at 12,000 rpm for 10 min at 4 °C. Finally, the supernatant was collected, and the protein concentration was determined with BCA kit according to the manufacturer's instructions. The protein preparation and LC-MS/MS detection using the iTRAQ technique were performed in Jingjie PTM Biolab Co. Ltd (Hangzhou, China).

### Antibodies and reagents

The anti-IFI16 antibody (ab55328) was purchased from Abcam (Cambridge, UK); the anti-FGF1 antibody (ab179455) was purchased from Abcam (Cambridge, UK); the anti-β-actin antibody (A5316) was purchased from Sigma (St. Louis, MO, USA).

### Plasmid construction and lentivirus infection

Short hairpin RNA (shRNA) sequences were cloned into the pSIH1-Puro vector (#26597, Addgene). The shRNA sequences for IFI16 were as follows: sh1, GGAAACTCTGAAGATTGATA, sh2, GATCATTGCCATAGCAAATTA. The full-length cDNA of human IFI16, FGF1 and FGF2 was cloned into the pLVX-IRES-Neo vector (632181, Clontech, CA, USA). Lentivirus were produced by HEK293-T cells and harvested 48 h after transfection, cells in which IFI16 was knocked down or overexpression were selected with 1 μg/ml puromycin or 200 μg/ml G418 for two weeks.

### Transwell assays

*In vitro*, cell invasion and migration assays were evaluated in 24-well Boyden chambers (Corning Incorporated, Corning, NY, USA). The wells were coated with or without Matrigel (BD Biosciences) and then incubated at 37 °C for 1 hour. Then, medium containing 20% FBS (680 μL) was added to each lower chamber, and the cells were placed in the upper chamber.

### Cell proliferation

Cells were seeded into a 96-well plate at a density of 1×10^3^ cells per well. Cell Counting Kit-8 (CCK-8) reagents (Dojindo, Tokyo, Japan) were added at a dilution of 1:10 to each well and incubated for 1h. The absorbance values were then measured at a wavelength of 450 nm using a microplate reader (BioTek, Winooski, VT, USA).

### Quantitative real-time PCR (qRT-PCR)

Total RNA was extracted from cells using TRIzol reagent and reverse transcribed using a QuantScript RT Kit (Tiangen, Beijing, China) in accordance with a standard protocol. QRT-PCR was performed on an ABI StepOnePlus Real-Time PCR System (Applied Biosystems, Foster City, CA, USA) using PowerUp SYBR Green Master Mix (Applied Biosystems). The following primers were used in the qRT-PCR assay: IFI16 (5′-TGAGCCCAAAGAGCAGAA-3′; 5′-GGTCAGCATTCACATCAGC-3′), IVL (5′-ACTGGCTCCACTTATTTCGGG-3′; 5′-GAGGTTGGGATTGGGGTCAT-3′), DHX58 (5′-GGTATCATCTTCACCCGCAC-3′, 5′-TCACTTCTTGCTGGTCCCTC-3′), ICAM1 (5′-AGCGGCTGACGTGTGCAGTAAT-3′, 5′-TCTGAGACCTCTGGCTTCGTCA-3′), BCAT1 (5′-TGTATCGCTCTGCTGTGAGG-3′, 5′-GGCTTTGGTAGGCTTCTTGAC-3′), ABCC2 (5′-CTCCTGTCTTCACCATCATCG-3′, 5′-AAAGGCACGGATAACTGGCAA-3′), FGF1 (5′-ACGGGCTTTTATACGGCTCA-3′, 5′-CCAACAAACCAATTCTTCTCT-3′), FGF2 (5′-GCTGTACTGCAAAAACGGGG-3′, 5′-AGCCAGGTAACGGTTAGCAC-3′), GAPDH (5′-CCGGGAAACTGTGGCGTGATGG-3′, 5′-AGGTGGAGGAGTGGGTGTCGCTGTT-3′).

### Animal experiments

Animal experiments were performed according to protocols approved by the Chinese Academy of Medical Sciences Cancer Hospital Animal Care and Use Committee. Male severe combined immune deficiency beige (SCID/Beige) mice (6 weeks old) purchased from Vital River (Beijing, China) were bred under specific pathogen-free conditions. SCID/Beige mice were used for the lung metastasis experiments.

For the lung metastasis model, equal number of control and IFI16-knockdown 30M cells were injected into the tail vein of SCID/Beige mice. After 45 days, these mice were injected with D-luciferin (15 mg/ml), and observed with the IVIS Lumina XRMS *in vivo* Imaging System (PerkinElmer, Waltham, MA, USA). Then, the mice were sacrificed and the lungs were harvested for bouin staining.

### Bioinformatics Methods in Proteomic Data

A total of 6,634 proteins (<1% FDR) were identified in the 30M/KYSE30 comparison group, of which 5,893 proteins contained quantitative information. The proteins which met the following criteria were confidently considered as differentially expressed proteins (DEPs): proteins showed an average ratio-fold change ≥1.5 or ≤1/1.5 and the same change trend in the three times experiments between the two cells (t-test; P < 0.05). In total, 383 DEPs were identified, including 295 up-regulated proteins and 88 down-regulated proteins (**Table S1**). The identified DEPs were then subjected to functional enrichment analysis, and the statistical significance of the functional enrichment was determined by Fisher's exact test.

### Bioinformatics Methods in RNA-Seq Data

The RNA-Seq assay was performed in IFI16-knocking down 30M cells by two shRNA (#1 and #2) and control cells to determine regulated genes by IFI16. The genes which met the following criteria were confidently considered as differentially expressed genes (DEGs): RNAs showed an average ratio-fold change ≥2 or ≤1/2 and the same change trend in the three times experiments between the two cells (t-test; *p*-adjusted < 0.05). We found that 98 genes were upregulated and 94 genes were downregulated in both shRNA #1 and #2 (**Table S2**). These DEGs were then subjected to functional enrichment analysis, and the statistical significance of the functional enrichment was determined by Fisher's exact test.

## Results

### The proteomic characteristics of 30M with high metastasis capacity

**Figure 1:**
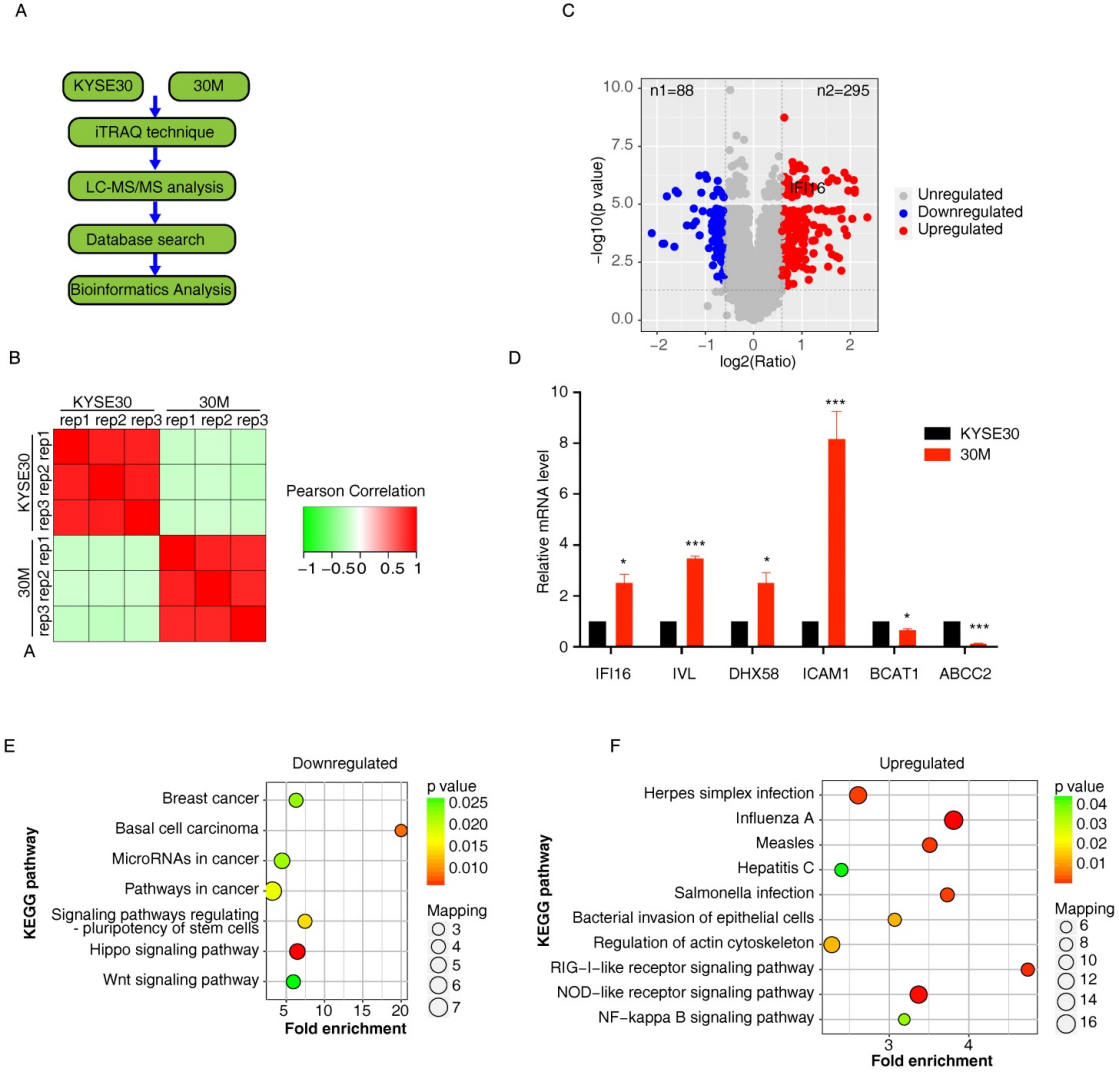
** iTRAQ analysis of highly metastatic 30M cell and the parental cells KYSE30. A.** Experimental design for the quantitative proteomics analysis. The proteins from KYSE30 and 30 M cells treated with iTRAQ technique and LC-MS/MS analysis. The differentially expressed proteins (DEPs) through database searches and bioinformatics analysis. **B.** Repeatability test between KYSE30 and 30M samples. **C.** Volcano plot showed the distribution of DEPs in the highly metastatic 30M cell compared with the parental cells KYSE30. The horizontal axis is the logarithmic value of the relative quantiative value of the protein after log2 conversion, and the vertical axis is the p-value after the -log10 conversion. The red dots indicate significantly up-regulated DEPs, and the blue dots indicate significantly down-regulated DEPs. **D.** Six random selected DEPs (upregulated DEPs: ICAM1, DH58, IVL, IFI16; downregulated DEPs: BCAT1 and ABCC2) in 30M/KYSE30 cells were confirmed by quantitative real-time PCR (qRT-PCR) (*p<0.05, **p<0.01, *** p<0.001). **E.** KEGG pathway enrichment analysis of downregulated DEPs that are significantly altered in 30M/KYSE30. **F.** KEGG pathway enrichment analysis of upregulated DEPs that are significantly altered in 30M/KYSE30.

A summary of the workflow for iTRAQ-based quantification of differentially expressed proteins (DEPs) is shown in **Figure 1A**. We selected a high metastasis capacity cell 30M and its parent KYSE30 control cells to perform proteomic analysis by iTRAQ technique in three replicate samples per group with consistent quality control (**Figure 1B**). A total of 6,634 proteins (<1% FDR) were identified in the 30M/KYSE30 comparison group, of which 5,893 proteins contained quantitative information. In total, 383 DEPs with statistical significance (p < 0.05 and fold change (FC) of T/N >1.5 or <1/1.5) were identified, including 295 up-regulated proteins and 88 down-regulated proteins (**Figure 1C** and **Table S1**). Quantitative real-time PCR (qRT-PCR) was performed to confirm the expression of 6 random selected DEPs in 30M/KYSE30 cells (**Figure 1D**).

The down-regulated DEPs were mainly localized in the plasma membrane (25%) and extracellularm (23%), and the up-regulated DEPs in 30M/KYSE30 was found that they were mainly localized in the cytoplasm (41%) and nucleus (29%). The KEGG pathway enrichment analysis demonstrated that the top 3 pathways enriched by downregulated DEPs in 30M/KYSE30 were Hippo signaling pathway, Basal cell carcinoma and signaling pathways regulating pluripotency of stem cells (**Figure 1E**). And top 3 pathways enriched by upregulated DEPs in 30M/KYSE30 were Influenza A, NOD-like receptor signaling pathway and RIG-I-like receptor signaling pathway (**Figure 1F**).

### IFI16 played a key role in ESCC metastasis

**Figure 2:**
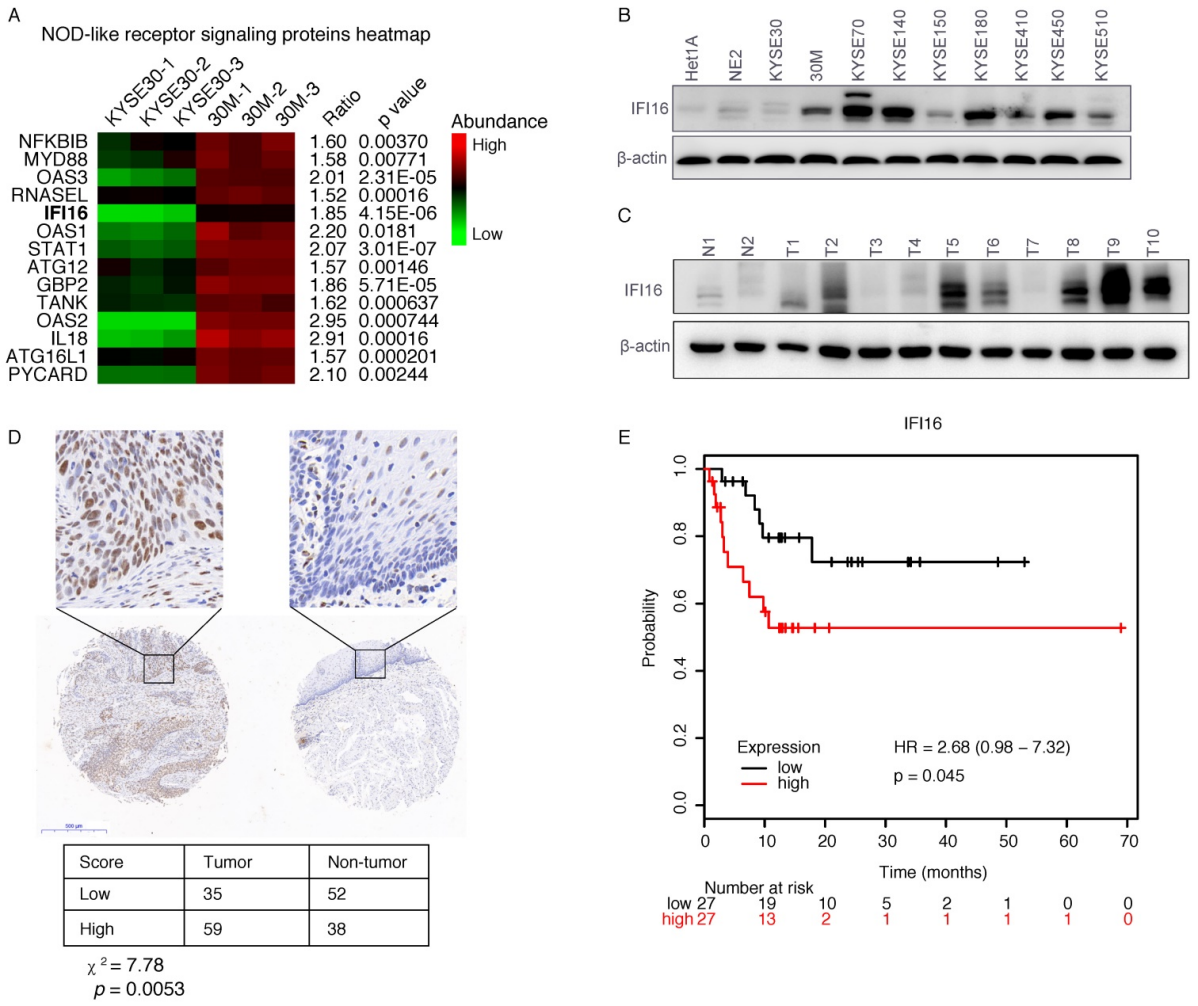
** IFI16 was overexpressed in ESCC tumor and predicted poor prognosis. A.** Heatmap of protein abundance of 14 DEPs in NOD-like receptor signaling pathway proteins. The colour represents the proteins abundance. **B.** The IFI16 protein levels were checked by western blot in ESCC cell lines. Among them, HET1A and NE2 are immortalized esophageal cells. **C.** A representative result of western blot shows the expression of IFI16 in the esophageal cancer tissues and normal tissues. **D.** Immunohistochemical staining for IFI16 expression in ESCC tissues and non-tumor tissue. Negative and weakly positive IFI16 were considered to represent low protein expression, while positive and strong IFI16 staining were considered to represent high protein expression. **E.** Kaplan-Meier analysis of the relationship between the expression of IFI16 and the survival time of relapse-free survival (RFS) in patients with esophageal cancer. The log-rank test p-values are shown.

Our proteomic data showed that IFI16, a molecule of NOD-like receptor signaling pathway was up-regulated in 30M/KYSE30 (FC=1.85, p=4.15×10^-6^) (**Figure 2A**), and the qRT-PCR and western blot results also confirmed this result (**Figure 1D** and **Figure 2B**). To evaluate the significance of IFI16 in ESCC development, we also detected the IFI16 proteins levels in different ESCC cell lines and ESCC tumor tissues by western blot. The result showed that IFI16 also increased in most of ESCC cells lines and ESCC tumor compared with immortalized esophageal cells (HET1A and NE2) and non-tumor tissue (**Figure 2B** and **Figure 2C**).

Next, to evaluate the clinical relevance of IFI16, we conducted further research on IFI16 through immunohistochemistry (IHC) assays, which produced similar results, with an increase expression of IFI16 in esophageal cancer tissues as compared with paracancerous tissue (**Figure 2D**). In addition, we also analyzed the relationship between IFI16 expression and the survival prognosis of esophageal cancer patients from the Kaplan-Meier (http://kmplot.com/analysis/) database. Compared with patients with esophageal cancer with low IFI16 expression, patients with high IFI16 expression had poorer relapse-free survival (RFS) (P=0.045) (**Figure 2E**).

**Figure 3:**
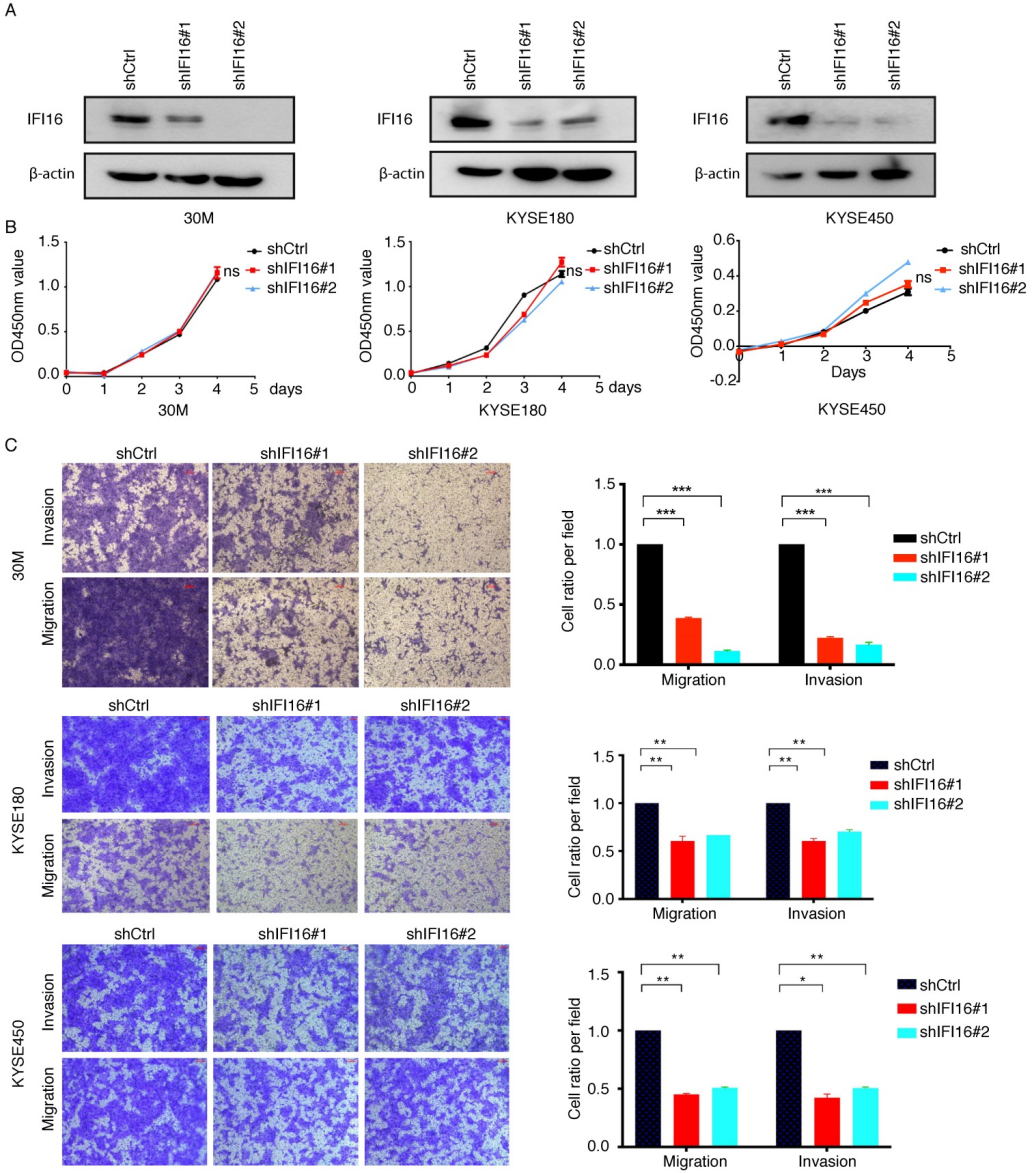
** Knocking down IFI16 inhibited ESCC cell migration and invasion *in vitro*. A-B.** The proliferation of IFI16 knockdown (shIFI16#1 and shIFI16#2) and control (shCtrl) cells were measured by CCK8 in 30M, KYSE180 and KYSE450 cells. ns: not significant. **C.** Representative images (left) and quantitative analysis (right) of migration and invasion assays in 30M, KYSE180 and KYSE450 cells. Scale bars=100 µm.

**Figure 4:**
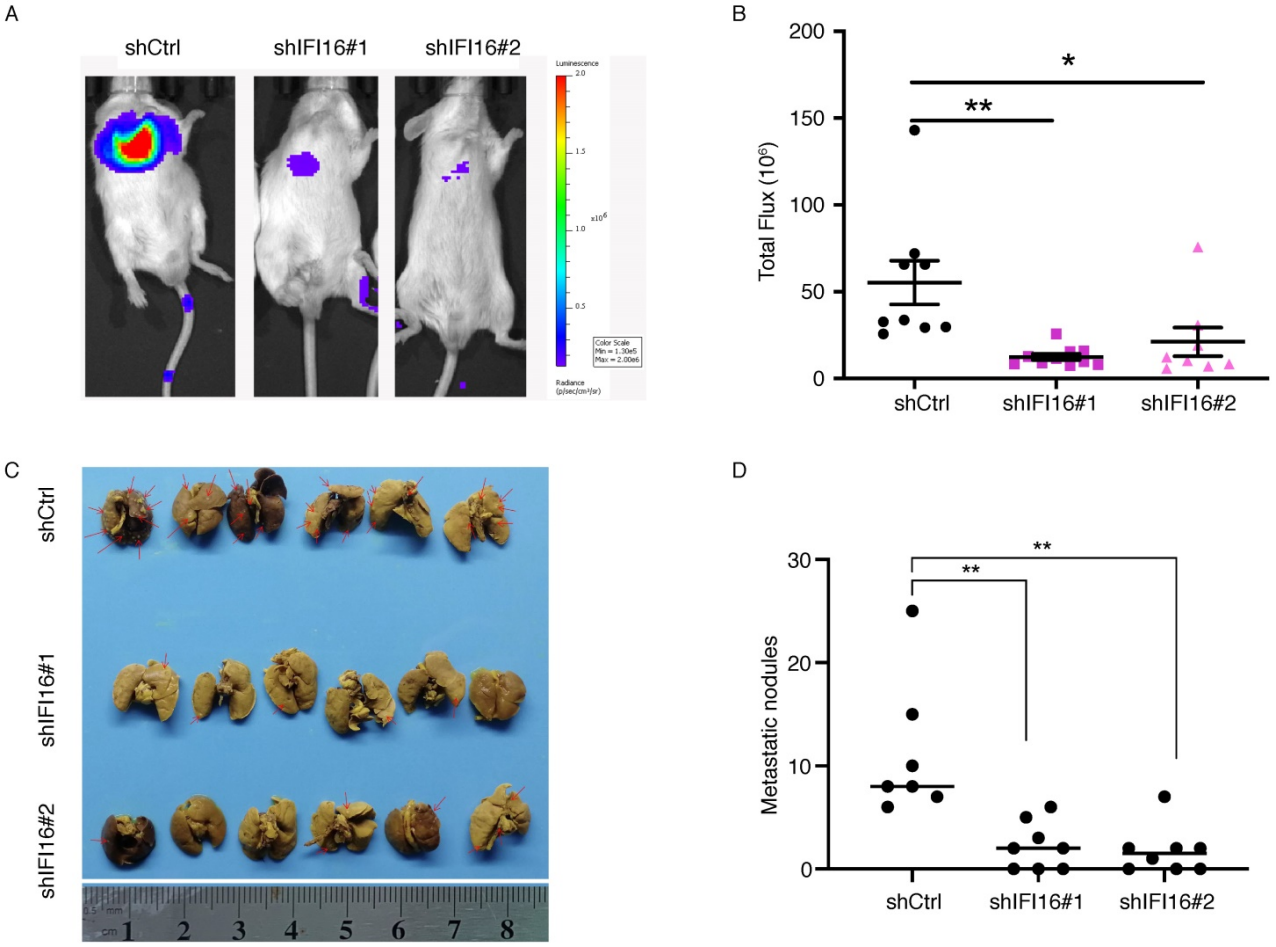
** IFI16 promotes lung metastasis *in vivo*. A.** Control cells and IFI16-knockdown 30M cells were injected into the tail vein of SCID/Beige mice and the lungs were harvested after 45 days for fluorescence. 30M cells labeled with luciferase in live imaging of small animals. The color scale describes the photon flux emitted by tumor cells. **B.** Results of quantitative analysis of fluorescence values (*p*<0.05). **C.** The lungs were harvested for bouin staining. **D.** Tumor nodules in lung tissues were counted (*p*<0.01).

The significant increase of IFI16 expression in the high metastatic potentials 30M cell and tumor tissues hinted us to investigate its role in metastasis regulation both* in vitro* and *in vivo*. We generated 30M, KYSE180 and KYSE450 cell lines with knockdown of IFI16 (**Figure 3A**). CCK8 assay showed that proliferation of 30M, KYSE180, and KYSE450 cells were not affected by IFI16 knockdown (**Figure 3B**). However, knockdown IFI16 markedly inhibited migration and invasion of 30M, KYSE180 and KYSE450 cells (**Figure 3C**). The results suggested that IFI16 plays a key role in increasing esophageal cancer cell migration and invasion *in vitro*. To explore whether IFI16 influences ESCC metastasis *in vivo*, we used a tail vein injection mouse models to evaluate the effect of IFI16. The number of lung nodules was significantly decreased by IFI16 knockdown in 30M cells (**Figure 4A-D**).

### IFI16 promotes ESCC metastasis through increasing FGF proteins expression

**Figure 5:**
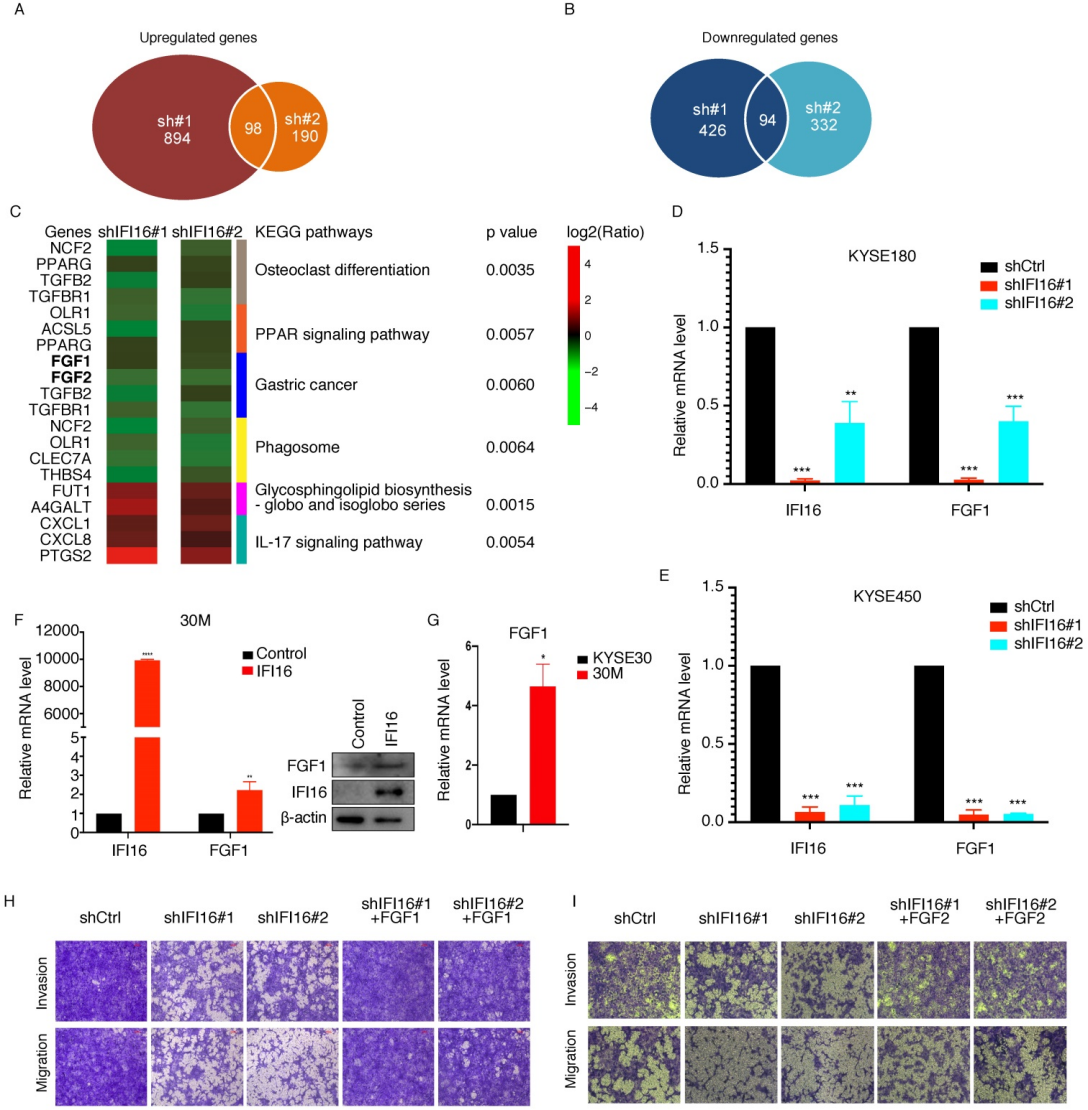
** IFI16 promoted the invasion and migration of ESCC cells through increasing FGF proteins expression. A.** Number of upregulated genes in both IFI16-shRNA#1 and IFI16-shRNA#2 cells. **B.** Number of downregulated genes in both IFI16-shRNA#1 and IFI16-shRNA#2 cells. **C.** Heatmap showing the genes expressing level ratio of shIFI16/shCtrl in top significant enriched KEGG pathway (p<0.01). **D-E.** IFI16 knockdown decreased the FGF1 gene expression in KYSE180 (D) and KYSE450 (E) cells. QRT-PCR analysis of FGF1 expression in IFI16-knockdown KYSE180 and KYSE450 cells. (*p<0.05, **p<0.01,*** p<0.001) **F.** IFI16 overexpression increased the FGF1 gene expression in 30M measured by realtime-PCR (left) and western blotting (right). **G.** FGF1 mRNA level was measured in KYSE30 and 30M cells by realtime-PCR. **H-I.** FGF1 (H) and FGF2 (I) overexpression rescued the inhibited migration and invasion ability by IFI16-depletion. The migration and invasion ability of FGF1 and FGF2 overexpression 30M-shIFI16 cells was analyzed by the transwell assay.

IFI16 located in the nucleoplasm and nucleolus is a member of the IFN-inducible HIN-200 gene family and as a regulatory factor that mediates the genes expression 11. To determine the underlying mechanism by which IFI16 promotes metastasis, we performed an RNA-Seq assay in IFI16-knocking down 30M cells by two shRNA (#1 and #2) and control cells, and the differentially expressed genes (DEGs) were further analysed (**Table S2**). After knocking down of IFI16 expression in both groups of sh#1 and sh#2, we identified 98 DEGs with upregulated expression and 94 DEGs with downregulated expression (**Figure 5A-B**). KEGG pathway enrichment analysis revealed that top 3 KEGG pathways enriched by upregulated DEGs were Glycosphingolipid biosynthesis, IL-17 signaling pathway and Bladder cancer and top 3 KEGG pathways enriched by downregulated DEGs were Osteoclast differentiation, PPAR signaling pathway and Gastric cancer pathway (**Figure 5C**). In addition, NOD-like receptor signaling pathway was also enriched by downregulated DEGs after knocking down IFI16.

Moreover, our RNA-Seq results showed that FGF proteins (FGF1, FGF2) were decreased in both IFI16-shRNA#1 and IFI16-shRNA#2 30M cells (**Figure 5C**). To further confirm that IFI16 can increase FGF1 expression in ESCC cells, we also knocked down IFI16 in KYSE180 and KYSE450 cells, and qRT-PCR results had showed that FGF1 also decreased in both two cells (**Figure 5D-E**). And overexpression of IFI16 could increase the FGF1 mRNA and protein level in 30M cell (**Figure 5F**). Moreover, FGF1 expression level was higher in 30M than in KYSE30 parent cell (**Figure 5G**). To further identify IFI16 promoted ESCC migration and invasion through increasing FGF gene expression, we overexpressed FGF1 and FGF2 in 30M cell after knocking down IFI16, and then measured the migration and invasion ability of cells. Our rescue experiment results showed that overexpression of FGF1 and FGF2 could rescue the inhibited migration and invasion ability by IFI16-depletion in 30M cells (**Figure 5H & I**).

## Discussion

In this study, we applied used iTRAQ combined with LC-MS/MS technology to compare the proteome of two esophageal cancer cells KYSE30 and 30M with the same genetic background but different metastatic potential, and further combined *in vivo* and *in vitro* experiments to identify the IFI16 protein related to esophageal cancer metastasis. Increasing evidence has extended the concept that chronic inflammation meditated by abberant NOD-like receptor signaling is a powerful driver of carcinogenesis 12, and served as the potential targets in cancer therapy 13. It was reported that chronic inflammation is associated with increased risk of ESCC development 14-16. IFI16, a component of NOD-like receptor signaling pathway, provides a broad surveillance role against viral DNA by promoting restriction of gene expression from the exogenous DNA and inducing innate immune responses 17 and upregulated in 30M cell with high metastasis capability. Our data had showed that IFI16 was also upregulated in ESCC tumors. Kaplan-Meier survival curve analysis showed that the relapse-free survival (RFS) of patients with high IFI16 level was significantly worse than that of patients with low IFI16 level. These results demonstrated that IFI16 played a key role in progress of ESCC tumors. *In vitro* transwell model and *in vivo* mouse model both showed that knockdown IFI16 remarkably decreased the metastasis ability of ESCC cells.

IFI16, also known as PYHIN, a transcriptional regulator, member of the PYHIN protein family that contains a pyrin domain and two DNA binding HIN domains 11, is involved in transcriptional regulation. MTA1 complex composed of metastasis-associated protein 1 (MTA1) and IFN-γ-inducible protein 16 (IFI16) contribute to the epigenetic regulation of genes expression 18. In addition, IFI16 regulates both self-renewal and differentiation gene expression during hESC trilineage specification through interaction with p53 19. To further clarify the mechanism of how IFI16 promoted ESCC metastasis, the RNA-seq analysis was performed in 30M cells by knockdown IFI16 by two shRNAs. The results showed that knocking down IFI16 could downregulate the expression of FGF proteins (FGF1, FGF2) expression. Similarly, we got the same result in KYSE180 and KYSE450 cells. The protein encoded by this gene is a member of the fibroblast growth factor (FGF) family. FGF family members possess broad mitogenic and cell survival activities, and are involved in a variety of biological processes, including cell proliferation, survival, migration, invasion, differentiation, and even angiogenesis 20. It was reported that FGF was also aberrantly expressed in a series of cancer, including pancreatic cancer 21, glioblastoma 22, gastrointestinal cancer 23 and ESCC 24. Deregulation of FGF signaling is implicated in both tumorigenesis and tumor invasion and metastasis 25. Moreover, overexpression FGF1 and FGF2 could rescue the migration and invasion ability of ESCC cells after IFI16 knockdown. Therefore, our results demonstrated that IFI16 promoted ESCC metastasis through increasing FGF proteins expressing.

In conclusion, our quantitative proteomics data in this study demonstrated that the IFI16 upregulated in highly metastatic ESCC cell played a key role in promoting the invasion and migration of ESCC cells through increasing FGF proteins expression.

## Supplementary Material

Supplementary table 1.

Supplementary table 2.
